# Mendel’s terminology and notation reveal his understanding of genetics

**DOI:** 10.1186/s41065-023-00276-x

**Published:** 2023-04-17

**Authors:** T. H. Noel Ellis, Peter J. van Dijk

**Affiliations:** 1 Department of Biochemistry and Metabolism, John Innes Centre, Norwich Research Park, Norwich, NR4 7UH UK; 2 Keygene N.V, Agro Business Park 90, 6708 PW Wageningen, The Netherlands

**Keywords:** Mendel, Terminology, Symbols, Genetic nomenclatutre, Olby

## Abstract

We describe both the terminology and use of symbols introduced by Mendel in his 1866 paper and discuss some misconceptions concerning their interpretation.

## Introduction

Mendel’s 1866 paper “Versuche über Pflanzen-Hybriden” set out to present a “generally applicable law governing the formation and development^1^ of hybrids” by determining the number and frequency of different forms in successive generations [19, 20]. To put this simply, Mendel sought to present a quantitative theory of inheritance. Many features of Mendel’s paper were innovative or at least unusual for his time. Not least of these was his introduction of new terminology and a symbolic representation of the nature of individuals, in terms of their potential offspring. As Stern wrote: "It [Mendel’s 1866 paper] does not simply announce the discovery of important facts by new methods of observation and experiment. Rather, in an act of highest creativity, it presents these facts in a conceptual scheme which gives them general meaning" [29]. Both Mendel’s genetic terminology and symbolic notation are, in essence, still in use today.

The most important terms used nowadays in genetic studies are phenotype, genotype, allele, homozygote and heterozygote. These genetic terms were coined in the early 1900s, after the death of Mendel, when there was an increased understanding of the wider applicability of his findings, and so they better reflect our current way of thinking. Mendel's understanding, however, must have been very different from ours, not least because reduction division (meiosis) and the distribution of the alleles of a diploid into haploid gametes were not known in his time. However, the processes he described are compatible with this, as they should be if they were to give a satisfactory explanation for inheritance.

Here we discuss these innovations of Mendel’s, and what they imply about his thinking concerning inheritance. We will also discuss how these have been interpreted by others, notably Heimans [13], Olby [23], Meijer [18], Campbell [7], Olby [24], Hartl and Orel [12], Orel and Hartl [26] and Muller-Wille and Orel [21]. Heimans [13] and Olby [23] have been highly critical of the genetic interpretation of Mendel’s work, which will be discussed at the end of this paper. Olby’s papers have been very influential and have received much attention in the history of science [5, 31], and education literature [1, 15], Campanile et al. [6, 28, 2], as well as in the secondary literature including popular science books (e.g. [11, 34, 16]). Indeed those critical of Olby's views have been described as subscribing to an ‘exceptionally inert myth’ [31].

### Mendel's terminology

Mendel wrote (in German) about "*Merkmale*", "*Charactere*", "*Factor*", and "*Elemente*". These words have a markedly different frequency and distribution in the 1866 article, indicative of their different meanings to Mendel. “*Merkmal* (plural *Merkmale*)” is by far the most frequently used (157 times) and appears throughout the article. The preferred translation of "*Merkmal*" is "character", rather than “trait”; Mendel's *Merkmale* have alternative states and are not quantitative differences. Sometimes Mendel used its synonym, “*Charactere*” (6 times). "*Factor*" is discussed only once, in the section on the fertilization cells. "*Elemente*" appears ten times in three consecutive paragraphs in the discussion; its usage in the paper is clearly not random.

Mendel introduced the terms dominance, "*dominirende*" and "*recessive*", recessiveness, for the state of expression of the character in the hybrid. The word “*dominiren”* was commonly used in German and had the meaning of ruling (*herschen*). “*Recessive*”, however, does not occur in German dictionaries of those days. Mendel introduced this term directly from Latin, where it means receding. As a priest, of course, Mendel was well-trained in Latin. We now apply dominant and recessive to alleles,^2^ but Mendel did not have of our concept of alleles so his use of "*dominant*" and "*recessive*" must have been different from ours. Mendel always used "*dominant*" or "*recessive*" in relation to "*Merkmal*" and "*Charactere*", never in direct combination with "*Factor*" or "*Elemente*".

Whether “*Charactere*” as used in the paper is to be understood in the modern senses of either a phenotype, or a genotype, depends on the context. It has been claimed that Mendel had no notion of a genotype [13, 23] and thought only about phenotypes, but in our view this is incorrect.

### Characters throughout the paper

Mendel specified "*Merkmal*" in four ways: “Parental-Character” (*Stamm-Merkmal*), “Hybrid-Character”, “Dominating Character”, and “Recessive Character”. The contrasting parent forms, e.g., round versus wrinkled seeds, had alternative Parental-Characters. Cultivation trials in 1854 and 1855 had shown that the Parental-Characters were true-breeding: they did not change in the next generation when self-pollinated. In modern genetic terms, these true breeding lines would be said to have a homozygous genotype at this particular locus, known as the *r* locus [3]. The same would be true of the other Parental-Characters Mendel studied. Pea cultivars then, as now, have a homozygous genotype at any given locus due to their mode of reproduction by self-fertilisation. Similarly wild peas are essentially true-breeding, with stable homozygous genotypes [14].

After 1856 Mendel started his experimental hybridizations. He used Hybrid-Character to designate the character of the progeny resulting from manual cross-fertilisation of contrasting parents. In modern terms this is the F1 hybrid, and Hybrid-Character designates the heterozygote. In the case of peas, due to dominance,^3^ each F1 Hybrid-Character is phenotypically identical to one of the two Parental-Characters. The Parental-Character that resembled the morphology of the F1 hybrid was called by Mendel the “Dominating Character”. Mendel noticed that when the hybrid was self-pollinated, the non-dominant Parental-Character reappeared in ¼ of the offspring. The non-dominant Parental-Character had not disappeared in the F1 hybid but was latent; Mendel denoted the non-dominant Parental-Character as the “Recessive-Character”.

Crucially, Mendel noticed that there was a difference between the dominant Parental-Character and the dominant Hybrid-Character although they appeared morphologically similar. The difference was that self-fertilization of the dominant Parental-Character exclusively generated plants with the same dominant Parental-Character, whereas self-fertilization of the dominant Hybrid-Character produced ¼ of the progeny with the recessive Parental-Character. For both of the seed characters Mendel studied, round vs wrinkled and green vs yellow cotyledons, plants with the dominant Hybrid-Character will have two types of seed in their pods,^4^ while plants with the dominant Parental-Character will have only one type of seed in their pods. Thus the plants with the dominant Hybrid-Character appear different from plants with the dominant Parental-Character, based on their progeny. This may be why Mendel mentioned “the development of the hybrid in their descendants” as the goal of the experiments in his introduction.

In his discussion of the generative cells (i.e. gametes), Mendel again referred to the 'dominant' or 'recessive’ characters. The gametes themselves are not tall or dwarf, round or wrinkled, yellow podded or green podded etc., so again Mendel's use of "*Merkmale*" refers to something other than the phenotype. Thus Mendel used a "*Merkmale*" to denote something akin to a genotype in several places, and akin to a phenotype in other places; he was aware of this distinction. In the context of the section on generative cells (gametes), Mendel used the words “*Factoren*” and “*Anlage*” which is further proof that he used "*Merkmale*" in this context in the modern sense, in the way we use alleles. When Heimans [13] and Olby [23] argue that Mendel did not think in genetic, but in phenotypic terms, this is only partially true. Given the lack of concepts like genotype, phenotype, and allele available to him at the time, it is hard to see how he could have been clearer in his 1865 lecture to an audience lacking any genetic background. Table 1 gives an overview of the these concepts and their relation to Mendel’s character types or symbols:

**Table 1:** Mendel's use of different "Merkmal" concepts. Mendel’s notations are compared with modern genotypic notations. Mendel used a short notation for plants as well as a longer one for the immediate products of fertilization

**Mendel**			**Modern**
Concept	Plant	Zygote (foundation cell)	
Parental-Character	A or a	A/A or a/a	AA or aa
Hybrid-Character	Aa	A/a	Aa
Dominating Character	A or Aa	A/A or A/a	AA or Aa
Recessive Character	a	a/a	aa

### Factors and their disposition in the fertilisation cells

The word "*Factoren*" (Factors) is used only once in BSHS section "The fertilisation cells of hybrids". In the introductory paragraph of this section Mendel wrote:

*" ... we find it confirmed everywhere that constant descendants can only be formed if the germ cells and the fertilising pollen are of the same kind and hence both are equipped with the ****disposition*** (Anlage)*** to animate**** completely identical individuals, as is the case with the normal fertilisation of pure species. We therefore have to consider it as necessary that, also in the generation of constant forms on the hybrid plant, completely identical factors* (Factoren) *act together."* (p24 our emphasis)

In this paragraph "constant descendants" and "constant forms" refer to what we would call homozygotes; individuals which, on selfing, breed true. The expression "completely identical" is alternatively translated as "exactly similar" by Drury and Bateson.

The word 'animate' (*beleben*) has other translations; create, vitalise, or vivify as noted in the BSHS translation. All of these words have a similar connotation of something that acts to initiate a (biological) process. This usage means that a factor is distinct from the process, or phenotype, that is brought about.

It is not surprising that in his discussion of the nature of the generative cells, Mendel needed to introduce a new word, “Factor”, because the nature of these cells is relevant to what may happen later when the generative cells fuse and the zygote is formed. The phenotype of the next generation develops; indeed the phenotype of a mature organism, and not of the generative cell itself. Factor invokes a sense of the potential to cause something to happen, rather than the thing which actually happens.

Mendel did not say what this animating principle was, what it was composed of, where it was to be found, whether it was numerous or how many of them would be found if one knew where to look. However, Mendel clearly stated that the factors in the male and female gametes are completely identical in pure breeding individuals, and in those progeny of hybrids with a “constant form”, i.e. homozygotes derived from a heterozygote.

### Cell elements in the concluding remarks

So far we have discussed content that Mendel most probably presented with his empirical results at the two lectures. In his second lecture about the reproductive cells Mendel will surely have mentioned factors. Later on, in the discussion, Mendel speculated about what was going on in the reproduction cells where he introduced the term “cell elements”. He used the word "*Elemente*" several times, but only in this one part of his "Closing remarks" where the discussion of the generative cells and their fate begins. This part of the paper very likely was not part of the two lectures [33], but was written between February 1865 and the summer of 1866 at the latest. In this section, Mendel contemplates what the nature of the (internal) factors and determinants that discriminated the gametes might be.

Modern scientific papers often summarize their results in a model that could explain the process under study. In his paper, Mendel provided a verbal model of the possible action of factors in the fertilizing cells. For this, he introduced the concept of “*Elemente*”.

"According to the opinion of famous physiologists, in phanerogams one germ and one pollen cell respectively unite to form a single cell*)^5^*that is able by absorption of matter and formation of new cells to ****develop**** itself further into an autonomous organism."* (p40, our emphasis)

This is close to our current understanding, but it misses the important ploidy difference between the gametes, which are haploid, and the zygote, which is diploid. This sentence is immediately followed by:

*"This ****development**** occurs according to a constant law, which is grounded in the material constitution and arrangement of the ****elements**** that attained a viable union in the cell."* (p41, our emphasis)

Here 'develop' and 'development' refer to the same process in the two sentences. The 'elements' are discussed further:

*"If the propagation cells are of the same kind and if they concur with the foundation cell of the maternal plant, then development of the new individual will be guided by the same law that is valid for the mother plant. If one succeeds in joining a germ cell with a pollen cell of different kind, then we must suppose that some compromise is taking place between those ****elements**** of the two cells that condition the mutual differences. The mediation cell emerging from this will form the foundation of the hybrid organism, whose ****development**** necessarily occurs according to a law other than that in the case of each of the two parent-species."* (p41, our emphasis)

Mendel did not speculate further about the nature of the elements. Possibly he thought of an analogy with chemical elements in which their grouping and nature in a compound also resulted into different properties and which could also be separated again. Mendel's aversion to too much speculation is evident from the comment he wrote a few years later on Darwin's speculative pangenesis theory: "*sich einem Eindrucke ohne Reflexion hingeben*" (to give in to an impression without reflection) [32]. It is often said that Mendel had a particulate theory of inheritance (for example, Wikipedia and many textbooks) but this does not mean that he explicitly stated that his elements were particles, like Darwin’s “gemmules” or De Vries’ “pangenes”.

It is clear, however that the elements were non-blending; they can be extracted intact from the hybrid; were discrete, atomic. It goes too far to say that Mendel thought about genes, but his elements come close to that.

A related concept appears four years later, in 1870, in Mendel’s ninth letter to Nägeli ([30] p97) where Mendel used the word “*Anlage*” when discussing sex determination in *Lychnis* (now *Silene*) plants, writing:

*“Is it chance only that the male plants occur here in the ratio 52: 203 or 1: 4, or has this ratio the same significance as in the first generation of hybrids [F2] with varying progeny?*^6^* I should doubt the latter, because of the strange conclusions which would have to be drawn in this case.* (translation [27]).

*On the other hand, the question cannot be dismissed so easily if one considers that the disposition* (Anlage) *for the functional development of either the pistil or the anthers had already to be determined by the organization of the zygotes*^7^*from which the plants emerged and that this difference in the zygotes might result from the both egg cells as well as the pollen cells being different in sexual disposition* (sex Anlage)*”* (our translation)*.*

Despite “the strange conclusions which would have to be drawn”, Mendel was not willing to give up his concept of different Anlagen in both the egg cells as well as the pollen grains. Thus Mendel strongly believed that the gametes contained different dispositions for different mature plant characters.

### Mendel’s innovative notation

Before Mendel it was usual to describe the general appearance (habitus) of a hybrid plant as a whole, as a “type”; de Candolle [10] designated two species, A and B and their hybrids as AB, meaning type AB. This concerned the individuals as a whole and not each of their various properties. At the same time as Mendel, Wichura [35] and Nägeli [22] used formulas to represent the repeated back-crossing of species hybrids with the parent species (e.g.,A-A-AB). This, too, referred to all distinct characters summed, and appears remarkably primitive compared to Mendel's annotation.

Mendel introduced a completely new notation, denoting the different traits with a different symbol. He used a capital letter, for example, "A", for one constant form of a trait, such as round-seeded, and a lowercase letter, "a" for the alternative form, wrinkled seeded. Mendel rendered the hybrid as “Aa”, referring to this particular trait alone, not to the plant as a whole. This was a revolutionary concept. In analyzing his F3 numerical results, Mendel probably recognized that the relative abundance of the two different types of true-breeding F2 individuals, (we would now call them homozygotes) and the F2 individuals which gave both types of progeny (heterozygous individuals), fitted the binomial theorem. This may have been the inspiration for him to develop a notation describing the “behaviour of the hybrids in the progeny”. The binomial theorem, (x + y)^2^ = x^2^ + 2xy + y^2^ (or xx + 2xy + yy) was well known in Mendel’s time and taught as part of mathematics at the Realschule. The unexpanded form was always given as (x + y)^2^ or (a + b)^2^. Mendel, however, used upper and lower cases: (A + a)^2^. His innovative notation, indicating the two alternative states, could capture dominance (upper case, A) and recessiveness (lower case, a), and was applicable to each of the individual characters of a plant. At times, Mendel used these symbols to describe plant traits, at other times he used them to describe the generative cells (gametes).

In Mendel’s paper an individual designated ‘Aa’ has the capacity to produce both ‘A’ and ‘a’ generative cells, even though phenotypically it appears like ‘A’, which cannot produce generative cells of type ‘a’. Importantly an ‘Aa’ individual does not produce ‘Aa’ gametes (generative cells). For Mendel there must have been something different between ‘A’ and ‘Aa’, they must differ in their development in some way such that one, but not the other, can produce ‘a’ generative cells (gametes).

Below we discuss Mendel’s use of symbols, what they imply about his thinking concerning inheritance and how these have been interpreted by others, notably Meijer [18], Campbell [7], Olby [24], Orel and Hartl [26], and Muller-Wille and Orel [21].

### Mendel’s use of symbols

Current usage in academic literature preserves Mendel’s innovation of using a single upper case letter to denote an element corresponding to the dominant character state and a single lowercase letter to represent the element corresponding to the recessive character state, but nowadays, two symbols are used for diploid organisms such as pea. This difference is far from trivial. In Mendel’s scheme, generative cells (haploid gametes) were designated by a single letter such as ‘A’ or ‘a’, just as they are today, but the plants from which they were derived could also be designated ‘A’, ‘a’ or ‘Aa’, rather than ‘*AA*’, ‘*aa*’ or ‘*Aa*’ as in modern usage. For Mendel, a plant designated ‘A’ has the ability to produce ‘A’ generative cells, ‘a’ denoted the ability to produce ‘a’ gametes and ‘Aa’ denoted the ability to produce both ‘A’ and ‘a’ gametes. ‘Aa’ individuals produced both ‘A’ and ‘a’ gametes in approximately equal numbers, and they did not produce ‘Aa’ gametes. Muller-Wille and Orel [21] commented that ‘it does not make much sense to annotate the product of the union of two cells of ‘like kind’ by a composite symbol’. Notably, Meijer [18] pointed out Mendel’s term ‘Aa’ refers to something invisible, manifest only in the next generation.

**Fig. 1:**
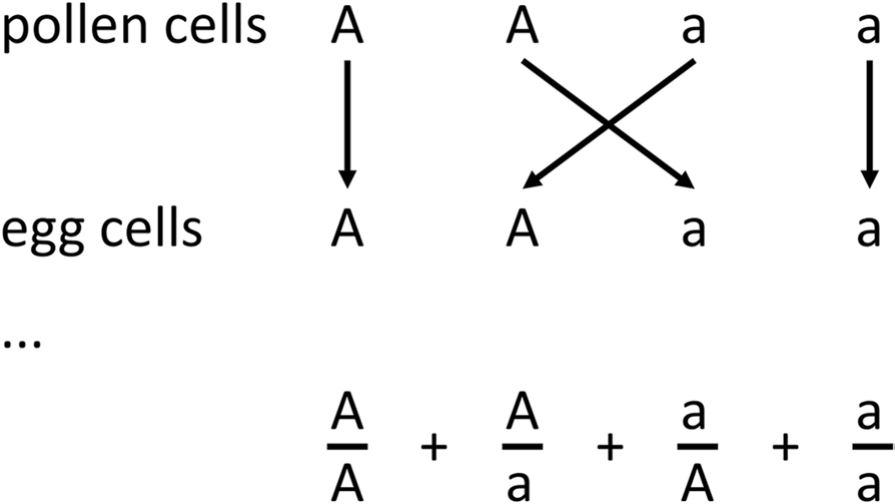
Mendel's explanation of the way that different germinal cells combine. The figure copies diagrams presented by Mendel in his 1866 paper. The ellipsis represents the following text, not included in the figure: “*The result of the fertilization may be made clear by putting the signs for the conjoined egg and pollen cells in the form of fractions, those for the pollen cells above and those for the egg cells below the line. We then have.*” (p30)

In his discussion of the germ cells Mendel presented a diagram (Fig. 1) which is relevant to this point:

At the end of the paragraph describing the figure Mendel presented a statement of equivalence:

$$\frac{\mathrm{A}}{\mathrm{A}}+\frac{\mathrm{A}}{\mathrm{a}}+\frac{\mathrm{a}}{\mathrm{A}}+\frac{\mathrm{a}}{\mathrm{a}}=\mathrm{A}+2\mathrm{Aa}+\mathrm{a}$$

The “fractional form” $\frac{\text{A}}{{\text{A}}}$ etc. in this equation refers back to the diagram illustrating the conjoined germ and pollen cells. This statement of equivalence makes clear the relationship between A, 2Aa and a, and the fractional forms $\frac{\text{A}}{{\text{A}}}, \frac{\mathrm{A}}{\mathrm{a}},\frac{\mathrm{a}}{\mathrm{A}}\mathrm{ and }\frac{\mathrm{a}}{\mathrm{a}}$, respectively. It should be emphasised that Mendel wrote "fractional form" not "fraction". This is a descriptive notation, not a mathematical assertion. Mendel's equivalence is not so surprising if we accept that 'A' etc. simply refers to the type(s) of gametes that can be produced (or identifies a type of gamete). Mendel’s scheme conveniently describes which types of gametes can be produced in what proportion, as well as explaining what phenotypes arise from the fusion of two gametes.

#### Criticisms of Mendel’s notation and segregation of homozygotes

Despite the fractional forms shown above, it has been argued by revisionists that Mendel's use of ‘A’ rather than ‘AA’, or ‘a’ rather than ‘aa’ suggests that Mendel had a flawed concept of the nature of the homozygote [23], see discussion in Orel and Hartl [25]), implying that he was not aware that there were two (indistinguishable) ‘A’s in homozygotes. Thus one is permitted to speculate that Mendel thought the ‘A’s from the two parents blended in the homozygote, and that for some reason blending did not happen in the ‘Aa’ hybrid.

Despite his clarity, Mendel has been criticised by Olby [23, 24] for his explanation of how the types of (haploid) generative cells are formed, quoting the sentence:

*"In the formation of these cells, all the elements participate in a totally free and uniform arrangement, while only the differing ones mutually exclude each other."* (BSHS p42)

as evidence that Mendel thought the identical elements do not mutually exclude one another. If, as we think, Mendel considered 'A', 'Aa' and 'a' to denote what types of generative cells can be produced, then the ‘exclusion’ of 'A' by 'A' etc. does not make sense. However, Mendel referred to ‘all the elements’ so was probably considering 'A' vs 'a', 'B' vs 'b', 'C' vs 'c' etc. Thus we can take this to mean that 'A' does not exclude 'B' or 'b' etc. and thus in current terminology the statement is about recombination, and not about segregation, as Olby implied.

With respect to the heterozygote Mendel wrote that ‘A’ and ‘a’ had a temporary compromise or mediation in the hybrid, writing a very significant paragraph in his concluding remarks:

*"With regard to those hybrids whose progeny are variable, one might perhaps assume that between the differing elements of the germ and pollen cell a mediation presumably occurs as well in so far as the formation of a cell serving as the foundation of the hybrid still becomes possible; yet that the compromise between the opposing elements is only a transient one and does not extend beyond the life of the hybrid plant. Since no changes in its habitus are perceptible during the whole vegetation period, we would have to conclude further, that the differing elements only succeed to step out of their enforced association during the development of the fertilisation cells. In the formation of these cells, all the elements participate in a totally free and uniform arrangement, while only the differing ones mutually exclude each other. In this way, the formation of as many kinds of germ and pollen cells would be enabled as there are combinations allowed for by the elements capable of development."* (BSHS p42)

The meaning of this is clear: the hybrid or heterozygote is uniformly so, no part of the plant is homozygous and exhibits the recessive parental phenotype, yet when it comes to the formation of the gametes the elements separate. This we can understand now as meiosis,^8^ that separates the alleles in the formation of the gametes, and this step is specific to the formation of gametes in male and female reproductive cells.

In the section about the "fertilisation cells of hybrids" Mendel had already written:

*"... as many different kinds of germ cells (germ vesicles) are formed in the ovary, and as many kinds of pollen cells in the anthers, as constant combination forms are possible, and that these germ- and pollen cells correspond in their inner constitution to the individual forms.*

*It is indeed possible to demonstrate along theoretical lines that this assumption would suffice to explain the development of hybrids in each individual generation, if at the same time one were allowed to presuppose that the different species of germ- and pollen cells are formed in equal quantity on average on the hybrid."* (BSHS p24)

This comment is precisely correct; in the absence of our present terminology and understanding of the underlying processes, it is hard to think of a better way of explaining what is happening.

Mendel's symbols can be seen to refer to what would eventually happen in the formation of gametes, whereas our current symbols refer to the actual state of the cell under discussion.

#### *Flower colour in* Phaseolus

A further criticism of Mendel related to his use of symbols comes from the discussion of his *Phaseolus* crosses. Mendel discussed two types of *Phaseolus* crosses. One was between *Phaseolus vulgaris* and *Phaseolus nanus* and the other between *Phaseolus nanus* and *Phaseolus multiflorus.* The first would now be considered an intra-specific cross within *Phaseolus vulgaris*, while the second is an inter-specific cross. *Phaseolus multiflorus* is a synonym of *Phaseolus coccineus*^9^ and this wider, inter-specific cross was problematic with poor fertility.

The F1 generation derived from the cross between *Phaseolus nanus* (with white flowers) and *Phaseolus multiflorus* (with purple flowers) behaved as was expected from the example of pea, but the F2 did not segregate 3:1.

*"The white flower and seed colour of Ph. nanus did indeed appear right away in the first generation* [we would call this the F2] *on a quite fertile specimen, but the remaining 30 plants developed flower colours which represented various shades from purple to pale violet."* (BSHS p33)

Mendel did not give up on this, but continued the experiment for several generations and concluded that.

*"… even these puzzling phenomena might probably be explained according to the law that is valid for Pisum, if one were allowed to presuppose that the flower and seed colour of Ph. multiflorus was composed of two or more entirely autonomous colours, each of which behaves individually in the same way as every other constant trait in the plant."* (BSHS p35)

We do not know how many genes affecting flower colour were segregating in this cross, but the appearance of one white-flowered F2 plant among 31 in total would be consistent with the segregation of recessive alleles of at least two distinct, genetically unlinked, genes, which in combination determine the recessive white condition. For two distinct loci the expectation is 1 in 16 (as opposed to one locus, where the expectation is 1 in 4, or three distinct loci where the expectation is 1 in 64).

Mendel's proposal was that the purple flower colour, A, was:

*" ... composed of autonomous traits A1* + *A2* + *. . . ., which evoke the overall impression of purple-red colouration, then by fertilisation with the differing trait of white colour a the hybrid conjunctions A1a* + *A2a* + *. . . . would have to be formed, and a similar situation would obtain with the corresponding colouration of the seed coat. According to the above presupposition, each of these hybrid colour-conjunctions would be autonomous and would accordingly develop entirely independently of the others. One then sees easily that from the combination of the individual developmental series an entire series of colours would have to arise. If, for example, A* = *A1* + *A2, then the hybrids A1a and A2a answer to the developmental series*

$$A_1+2A_1a+a$$

$$A_2+2A_2a+a"$$

and this gave 9 combinations of generative cells, which would produce 4 different types of constant progeny and 5 different types of variable progeny, heterozygous at *A1*, *A2*, or both. This bifactorial series had already been shown in the paper, when Mendel described pea hybrids with two differentiating characters using the notation 'A', 'a' and 'B', 'b' (instead of 'A1,a' and 'A2,a') and also in his discussion of the reproductive cells of the hybrid resulting from a bifactorial cross, which was presented as “fractional” forms, shown, with some rearrangement, in Table 2:

**Table 2:** Mendel's representation of zygotic ratios in a bifactorial cross

		$\frac{\mathrm{AB}}{\mathrm{AB}}$	+	2	$\frac{\mathrm{AB}}{\mathrm{aB}}$	+		$\frac{\mathrm{aB}}{\mathrm{aB}}$
+	2	$\frac{\mathrm{AB}}{\mathrm{Ab}}$	+	4	$\frac{\mathrm{AB}}{\mathrm{ab}}$	+	2	$\frac{\mathrm{aB}}{\mathrm{ab}}$
		$\frac{\mathrm{Ab}}{\mathrm{Ab}}$	+	2	$\frac{\mathrm{Ab}}{\mathrm{ab}}$	+		$\frac{\mathrm{ab}}{\mathrm{ab}}$

The way that Mendel laid out his table for the *Phaseolus* traits A_1_ and A_2_ (Table 3) below is clearly similar to Table 2 above, indicating that he was thinking in a similar way. The extended tabulation for the *Phaseolus* flower colour case would be as shown in the uppermost panel of Table 3. Mendel’s actual tabulation (bottom panel, Table 3) differs only in that the subscripts are missing from the lower case ‘a’s and the reversal of the order of symbols in the rightmost column:

**Table 3:** Tabulation of Mendel's *Phaseolus* cross

Extended tabulation of expected zygotic ratios in Mendel’s *Phaseolus* cross								
		$\frac{{\mathrm{A}}_{1}\;\;{\mathrm{A}}_{2}}{{\mathrm{A}}_{1}\;\;{\mathrm{A}}_{2}}$	+	2	$\frac{{\mathrm{A}}_{1}\;\;{\mathrm{A}}_{2}}{{\mathrm{a}}_{1}\;\;{\mathrm{ A}}_{2}}$	+		$\frac{{\mathrm{a}}_{1}\;\;{\mathrm{A}}_{2}}{{\mathrm{a}}_{1}\;\;{\mathrm{ A}}_{2}}$
+	2	$\frac{{\mathrm{A}}_{1}\;\;{\mathrm{A}}_{2}}{{\mathrm{A}}_{1}\;\;{\mathrm{a}}_{2}}$	+	4	$\frac{{\mathrm{A}}_{1}\;\;{\mathrm{A}}_{2}}{{\mathrm{a}}_{1}\;\;{\mathrm{a}}_{2}}$	+	2	$\frac{{\mathrm{a}}_{1}\;\;{\mathrm{A}}_{2}}{{\mathrm{a}}_{1}\;\;{\mathrm{a}}_{2}}$
		$\frac{{\mathrm{A}}_{1}\;\;{\mathrm{a}}_{2}}{{\mathrm{A}}_{1}\;\;{\mathrm{a}}_{2}}$	+	2	$\frac{{\mathrm{A}}_{1}\;\;{\mathrm{a}}_{2}}{{\mathrm{a}}_{1}\;\;{\mathrm{a}}_{2}}$	+		$\frac{{\mathrm{a}}_{1}\;\;{\mathrm{a}}_{2}}{{\mathrm{a}}_{1}\;\;{\mathrm{a}}_{2}}$
Which Mendel explained is equivalent to:								
	1	${\mathrm{A}}_{1}\;\;{\mathrm{ A}}_{2}$		2	${\mathrm{A}}_{1}{{\mathrm{a}}_{1}\;\;\mathrm{ A}}_{2}$		1	${\mathrm{a}}_{1}\;\;{\mathrm{ A}}_{2}$
	2	${\mathrm{A}}_{1}\;\;{\mathrm{ A}}_{2}{\mathrm{a}}_{2}$		4	${\mathrm{A}}_{1}{\mathrm{a}}_{1}\;\;{\mathrm{A}}_{2}{\mathrm{a}}_{2}$		2	${\mathrm{a}}_{1}\;\;{\mathrm{ A}}_{2}{\mathrm{a}}_{2}$
	1	${\mathrm{A}}_{1}\;\;{\mathrm{a}}_{2}$		2	${\mathrm{A}}_{1} {\mathrm{a}}_{1}\;\;{\mathrm{a}}_{2}$		1	${\mathrm{a}}_{1}\;\;{\mathrm{a}}_{2}$
Mendel’s actual tabulation:								
	1	${\mathrm{A}}_{1}\;\;{\mathrm{ A}}_{2}$		2	${\mathrm{A}}_{1}{\mathrm{a}}\;\;\mathrm A_{2}$		1	${\mathrm{A}}_{2}\;\;a$
	2	${\mathrm{A}}_{1}\;\;{\mathrm{ A}}_{2}\mathrm{a}$		4	${\mathrm{A}}_{1}\mathrm{a}\;\;{\mathrm{A}}_{2}\mathrm{a}$		2	${\mathrm{A}}_{2}\mathrm{a\;\;a}$
	1	${\mathrm{A}}_{1}\;\;\mathrm{a}$		2	${\mathrm{A}}_{1}\mathrm{a}\;\;\mathrm{a}$		1	$\mathrm{a\;\;a}$

The objection raised by Olby and Mayr [17, 23] is that Mendel used the symbol 'a' where we might have expected ‘a_1_’ and ‘a_2_’.

"*What seems odd in Mendel's treatment of the Phaseolus data is his failure to explain why he made no apology for putting both A1 and A2 with the same contrasted character a. ...*

The chief reason for this obscurity was, we believe, that Mendel was thinking in terms of ***the white colour*** when he wrote down ***a***. ... If he had been talking about hereditary factors he would have recognised that the a which is distinguished from A1 must be physically distinct and independent though not phenotypically distinct from that which is contrasted with A2" [23], his ***emphasis***).

Olby continued (again with Olby's ***emphasis***):

"This shows that ***he did not*** in his mathematical formulation of his experiments ***go from the character pair to the pair of factors***. His treatment of flower colour in Phaseolus suggests that he ***was not even thinking of the pair of mutually excluding factors*** or the Allel (Bateson's Allelomorph)."

The sense throughout, however is the same as that in other parts of the paper, namely the ‘addition’ of germ cells of specified types as in the ‘fraction’ a/a. Thus Olby appears to be suggesting that either (and possibly both) A_1_ and A_2_ are alternatives to the same a, in some three-way association. If Olby’s reasoning is correct, then it is clear why he thought Mendel was no Mendelian i.e. had no concept of mutually excluding character states. However, dismissing ‘a a’ as being like the fractional form $\frac{\text{a}}{{\text{a}}}$ as shown in Fig. 1 is a conjecture which is not well supported.

Mendel wrote that the flower colour ‘A’ would be supposed to be composed of the autonomous traits A_1_ + A_2_ + … This is a description of the phenotype, the flower colour, and a statement that it is a combination of (sub-)traits. What Mendel needed to explain was how such a combination can explain segregation in the F2 where:

"*… from the conjunction of white and purple-red colouration a whole range of colours from purple to pale-violet and white emerges*" (p33)

**Fig. 2:**
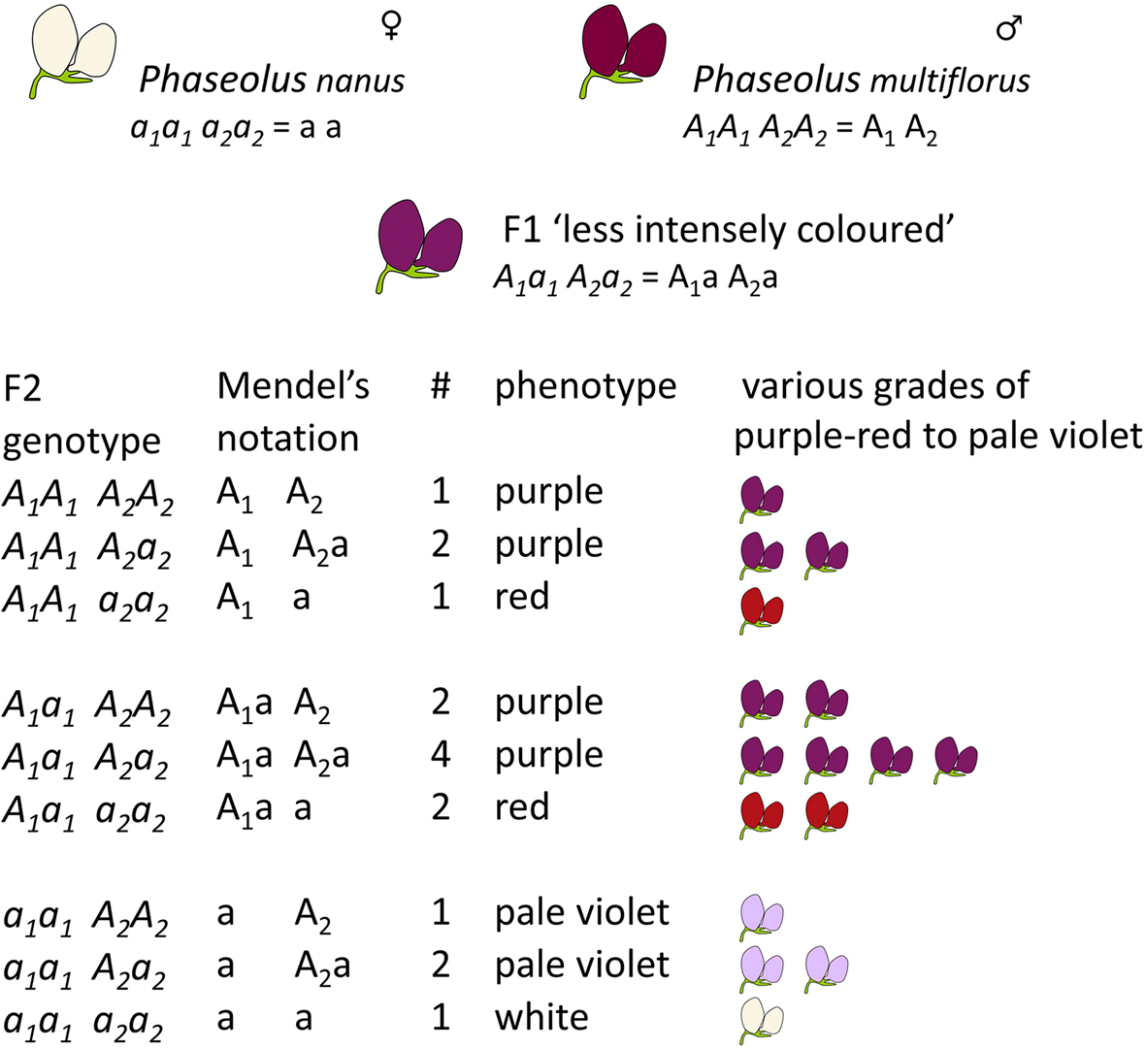
Mendel’s proposed explanations of flower colour variation in the F2 progeny of his *Phaseolus* cross. Columns represent the genotype and phenotype where the genes A1 and A2 determine flower colour. In Mendel’s model A1 and A2 combine to give various colours

‘A’ corresponds to purple-red conditioned by A_1_ + A_2_ …, but A_1_ + a or a + A_2_ condition other colours such as purple or pale violet etc. Mendel did not specify what colour corresponds to A_1_ or A_2_ alone, but Mendel's model, presented in modern terms, would look like something along the lines shown, in Fig. 2.

Mendel’s model accounts for four colours and has the 9:3:3:1 segregation ratio that he had described earlier. Mendel considered that a more complex range of colours could be brought about with additional factors. The way the symbols are aligned in Mendel's notation (Table 2 and Fig. 2) allows the symbol 'a' to occur in different vertical positions, and it appears twice in the four combinations: A1a A2a; A1a a; A2a a and aa.

Olby’s [23] proposal, that "Mendel was thinking in terms of *the white colour* when he wrote down *a*" (see also [9]) is not clearly defined.

We could ask why Mendel did not use symbols like R for red, V for violet etc., but used instead, A_1_, A_2_, with the explanation that A_1_ + A_2_ + … condition purple-red. Mendel was not fixed on a two-factor model, but used A_1_, and A_2_ as a simple example. He may have thought about more complicated models, where A_1_ + A_3_ would give a different colour from A_1_ + A_2_ (as is necessary if there are to be many different colours in the F2) in which case it is difficult to define what A_1_ actually confers. A_1_ and A_2_ seem to refer to pigments that may be combined, much as water colour pigments may be combined. Alternatively, it is also possible that A_1_, A_2_ etc. were intended to represent transformations of the colour of a pigment for example, as a pH changes the colour of an indicator.

For Mendel the difference between A and Aa was whether A bred true or not. Mendel may have considered 'a' to be like zero, or 'nothing'. This allows the formulation {A_1_ and nothing} + {A_2_ and nothing} as equivalent to the phenotypes A_1_ + A_2_, but 'A' alone does not correctly describe the types of generative cells A1a or A2a can produce. This way of thinking about ‘a’ is like a deletion allele. Today we would write the double heterozygote *Aa Bb* if *a* is a deletion allele of *A* and *b* is a deletion allele of *B.* We do this despite the fact that *a* and *b* are identical; they are both nothing. In this case, *a* and *b* are used just to keep track of what is missing.

Mendel knew that if A_1_ was present in a particular plant, the only way to determine whether A_1_ is or is not true breeding is to look in the next generation. If the plant is A1a it is pigmented and it is not true breeding, but if it is A1 it is pigmented and it is true breeding. We do not know what colour A_1_ corresponds to, because the final colour also depends on A_2_ or A_3_ etc.

For Mendel then, 'a' need not determine any colour, its only effect is whether or not the colour breeds true. If A_1_ is to denote a particular colour, or contribution to colour, then this is something that 'a' does not do and hence distinguishing between a_1_ and a_2_ would be a distinction between two inactions.

It appears that Mendel was indeed using 'a' like the use of zero in Arabic numerals where the two zeros in 100 have a different meaning even though both denote nothing; it is their relative positions that give them meaning. If Mendel had used a_1_ and a_2_ in this context it would have been a very clear demonstration that he was thinking about the problem exactly as we would. The alignment of the 'a's in Mendel’s table and their replication in some cases is very close to our thinking, so much so that it is tempting to assume that Mendel was thinking as we do, but that is going too far, because some of our concepts, such as haploid gametes, were not available to him.

What seems remarkable is that despite observing a multitude of colours, Mendel came to the conclusion that the segregation of flower colour in *Phaseolus* is explained in exactly the same way as for other factors that segregate 1:2:1.

Even though the *Phaseolus* cross in which flower colour segregated was confounded by sterility, violating one of his initial conditions for a valid experiment, Mendel used *Phaseolus* flower colour as an example of a challenge to his theory that could be met. In a statement demonstrating his rigorous thinking; he wrote:

*"It must, nevertheless, not be forgotten that the explanation here attempted is based on a mere hypothesis, only supported by the very imperfect result of the experiment just described."*

## Conclusion

The terminology of dominance and recessiveness that Mendel introduced is still in use but, his four types of ‘character’, though easily understood, have been replaced by descriptions of allelic states. Mendel used symbols in a variety of ways to keep track of how his factors (characters or elements) behaved in the offspring of crosses and in the selfed progeny of hybrids (heterozygotes). Mendel’s symbols are superficially similar to modern usage, but served a different function; they predicted what types of generative cells (gametes) would be produced and provided the rules by which these could be combined. Modern usage describes the allelic state of an individual, and from this we can use our understanding of meiosis to predict the types of gametes an individual is capable of generating. Current usage is descriptive while Mendel’s notation was predictive; our symbols are closer to a phenotypic description than Mendel's.

## Data Availability

Not applicable.

## Footnotes

1 The word “development” here refers to the behaviour of the hybrid over successive generations.

2 The dominant character state is seen in a heterozygote and noted as present, while the recessive character state is not seen in a heterozygote, but is seen in the next generation, in the progeny of a heterozygote. The recessive character state is therefore noted as absent, hidden, or receding, and can only be revealed in subsequent generations. The important point is that the recessive allele itself is present in a heterozygote; the presence/absence refers to the observable character state, not to the allele, so our current usage of recessive is really a shorthand for ‘the allele which confers the recessive phenotype’.

3 Rather, it is because the Hybrid-Character resembles one of the two Parental-Characters that Mendel used the term dominance to descripe this behaviour.

4 The seed coat is maternal tissue and therefore of the same generation as the plant on which it is borne. The embryo inside the seed coat is of the following generation, derived from the zygote formed by the fusion of the egg cell and the sperm cell. This embryo and the plant which grows from the embryo, are of the same generation. Once a pea seed is planted in the soil it becomes hydrated and its maternal seed coat splits open, eventually rotting away. The seedling matures and produces ovule coats inside its pods, that are renamed seedcoats once they envelop the embryos of the following generation. The relevant generation for a seed character depends on the character being investigated. The endosperm is different again, but this disappears early in pea seed development.

5 At this position Mendel referred to the one footnote in the 1866 paper.

6 Sex determination in *Silene* is of the XX / XY system (Blackburn 1923), but interestingly Mendel did not find a 1:1 segregation of the sexes as might be expected, but found instead a 1: 4 male: female ratio (52 male to 151 female). It was later dicovered that the biased sex-ratio is caused by Y-bearing pollen being competitively inferior to X pollen in the fertilization of egg cells. Less dense pollination, with less intense pollen tube competition, results in fewer than expected XY individuals [8].

7 The term “zygote” (like homozygote) was coined in 1878 by Eduard Strasburger. Mendel used instead “ground cell” (Grundzelle).

8 The importance of the reduction division in meiosis was first understood by Weismann in 1892, almost 30 years later.

9
http://www.theplantlist.org/tpl1.1/record/ild-2930
